# Trends in gastroenteritis-associated mortality in the United States, 1985–2005: variations by ICD-9 and ICD-10 codes

**DOI:** 10.1186/s12876-014-0211-0

**Published:** 2014-12-10

**Authors:** Jyotsna S Jagai, Genee S Smith, Judith E Schmid, Timothy J Wade

**Affiliations:** School of Public Health, Division of Environmental and Occupational Health Sciences, University of Illinois at Chicago, Chicago, IL USA; Gillings School of Global Public Health , Department of Epidemiology, University of North Carolina at Chapel Hill, Chapel Hill, NC USA; Office of Research and Development, National Health and Environmental Effects Research Laboratory, Research Cores Unit, U.S. Environmental Protection Agency, Research Triangle Park, NC USA; Office of Research and Development, National Health and Environmental Effects Research Laboratory, Environmental Public Health Division, Epidemiology Branch, U.S. Environmental Protection Agency, Research Triangle Park, NC USA

**Keywords:** Gastroenteritis, Mortality, Intestinal infections

## Abstract

**Background:**

Trends in gastroenteritis-associated mortality are changing over time with development of antibiotic resistant strains of certain pathogens, improved diagnostic methods, and changing healthcare. In 1999, ICD-10 coding was introduced for mortality records which can also affect trends. We assess trends in gastroenteritis-associated mortality and changes associated with coding.

**Methods:**

Trends in gastroenteritis-associated mortality rates in the United States were examined using the National Center for Health Statistics Multiple Cause-of-Death Mortality databases for 1985–2005. All deaths with the underlying cause or any contributing cause included gastroenteritis were included. Cases were selected based on ICD9 (pre-1999) and ICD10 (1999–2005) codes and all analyses were stratified by ICD usage. Annual trends in age adjusted mortality rates were assessed using linear regression spline analysis. Relative risks and 95% confidence intervals (CIs) were calculated using Poisson regression adjusted for age group, sex, race, and region.

**Results:**

There were a total of 190,674 deaths related to gastroenteritis in the U.S. from 1985–2005 with an average of 9,080 per year. During this time the percent of deaths related to gastroenteritis more than tripled, increasing from 0.25% to 0.80% of all deaths. Though the time periods varied in length, we demonstrate a significant increase in slope from a 0.0054% annual increase during the period 1985–1998, when ICD-9 coding was used, to a 0.0550% annual increase during 1999–2005, when ICD-10 coding was used. For both time periods, the oldest age group (75+ years) demonstrated the highest risk of death due to gastroenteritis. Additionally, males demonstrated higher risk than females and blacks were at higher risk than whites for death due to gastroenteritis.

**Conclusions:**

This analysis demonstrates the public health burden of gastroenteritis-associated mortality in the United States and changes in trends due to change from ICD-9 to ICD-10 coding. The overall rate of gastroenteritis-associated mortality has more than tripled over the 21-year period from 1985 to 2005 and the primary burden of deaths due to gastroenteritis is in the elderly population.

## Background

Worldwide, gastrointestinal infections are a major, and often preventable, cause of mortality. In much of the developing world, mortality due to gastrointestinal infections disproportionately impacts children and is often associated with poor hygienic conditions (e.g., contaminated food or water and person-to-person transmission) [1]. In contrast, in the United States the elderly have a higher mortality rate due to gastrointestinal infections [2,3]. Trends in gastroenteritis-associated mortality are changing over time [4]. These changes may be due to several factors including the development of antibiotic resistant strains of certain pathogens, changing healthcare practices, the prevalence of immunosuppressive conditions, and demographic changes such as a growing elderly population.

Rates of enteric infection and mortality due to enteric infections steadily dropped during the 20th century due to disinfection of drinking water and improved hygiene practices. However, in the 1990s and early 2000s, mortality rates in the United States began to increase [2,3]. This is consistent with a rising proportion of Americans at increased risk for severe consequences due to enteric infections, including the elderly and those that are immunocompromised [5,6]. Data from the Foodborne Diseases Active Surveillance Network (FoodNet), which collects data for 10 U.S. states, together with passive surveillance data were used to estimate that 31 major pathogens cause 1,351 deaths (90% CrI 712–2,268) annually [7]. In addition, an estimated 1,686 deaths (90% CrI 369–3,338) annually are due to foodborne illness from unspecified pathogenic agents [8]. This represents only a fraction of all deaths due to enteric infections which can also occur through waterborne and person-to-person transmission. Improved diagnostics, accuracy, and completeness of coding for deaths due to enteric infections may be causes for the demonstrated increasing trend.

Mortality records are coded using the International Classification of Disease (ICD), which was recently modified. In 1999, coding for mortality changed from using the ICD-9 scheme to ICD-10. The ICD-10 system is more detailed with approximately 8,000 categories for disease classification compared to about 5,000 categories in the ICD-9 system [9]. Few studies have demonstrated that the changes from ICD-9 to ICD-10 coding can affect trends in mortality. A study in Italy demonstrated that there was little variability between the two schemes for the larger disease groups, such as diseases of the circulatory system, however, the variability was higher for ‘minor’ disease groups such as infectious diseases and respiratory diseases [10]. A study focusing on respiratory disease mortality in the United Kingdom demonstrated that there was a 22% decrease in deaths assigned to respiratory disease under the ICD-10 coding scheme [11]. In the Southeastern United States, a study found that the change to ICD10 underestimated mortality due to heart disease and cerebrovascular disease and overestimates deaths due to diabetes [12]. In the U.S. it has been shown that implementing ICD-10 has variable effects on the discontinuity in trend; for some leading causes of death, such as influenza and pneumonia the discontinuity is substantial [13].

In this study we examine rates and trends in gastroenteritis-associated mortality for a 21-year period from 1985 to 2005, during which the ICD-10 coding scheme, was implemented by categories of pathogens (viral, bacterial, protozoal). The ICD-10 coding scheme differs in several aspects from the ICD-9 scheme, including more detailed classification. Of importance for this study, coding rules and rules for selecting the underlying cause of death have been changed in the ICD-10 system [13]. Therefore, in this paper our analysis is stratified based on the ICD system used and assesses changes in ICD coding on gastroenteritis associated mortality.

## Methods

### Data

We conducted a population-based, descriptive study of data from the National Center for Health Statistics (NCHS) Multiple Cause of Death Mortality database for the years 1985–2005 [14]. We utilized individual level data for which we obtained a data use agreement from NCHS. The data use agreement was only for the period 1985–2005 therefore, we were not able to consider out years. Each entry includes demographic and residence information for the decedent as well as the underlying cause of death and up to 20 contributing conditions which are listed on the death certificate. The cause of death and conditions are coded using the International Classification of Diseases (ICD) codes. We abstracted all deaths for which the underlying cause or any contributing cause of death included gastrointestinal infection. Deaths prior to 1999 were coded using the International Classification of Diseases, Ninth Revision (ICD-9) codes. We abstracted records with the following codes listed for underlying cause or a contributing cause of death: 001–009, 041, 047, 070, 074, 079, 127, and 558. From 1999–2005, deaths were coded using the International Classification of Diseases and Related Health Problems, Tenth Revision (ICD-10) codes. We selected the records with the following gastrointestinal conditions listed: A00.0-09, A08, A87, B15, B08.4, B34.1, B77-79, B81-82, B95-97, and K52.

We calculated cause-specific mortality rates for gastrointestinal illness from 1985 – 2005 by grouping deaths into five broad pathogen categories (bacterial, viral, protozoal, unknown noninfectious, and unknown infectious). The specific ICD-9 and ICD-10 codes included in each category are provided in Table 1. For the bacterial group, analysis was considered with and without records of deaths due to *Clostridium difficile* (ICD9: 008.45, ICD10: A04.7) because this infection contributed 51.7% of cases and will drive the results for this category. Population estimates for each year were obtained from the U.S. Bureau of Census [15] and were used to calculate rates. The mortality data from the NCHS was linked to population estimates from the Census Bureau by age, race, gender, and regions. Race was categorized as White, Black, and Other. Age was classified into 8 increments (0–4, 5–24, 25–34, 35–44, 45–54, 55–64, 65–74, and over 75. Region was defined by the U.S. census as Northeast, Midwest, South, and West [15]. All deaths occurring outside of the United States were excluded from the analysis.

**Table 1:** **Specific ICD-9 and ICD-10 codes included in each category of gastroenteritis-associated mortality**

	**ICD-9**	**ICD-10**
**Bacterial**	001-005, 008.0-008.5, 041	A00.0, A01.00, A01.1-A01.4, A02.0-02.1, A02.20, A02.8-A02.9, A03.0-A03.3, A03.8-A03.9, A05.0-A05.2, A05.8-A05.5, A05.8-A05.9, A04, A28.2, A49.1-A49.3, A49.9, B95.0-B95.8, B96.1-B96.7, B96.81-B996.82, B96.89
**Viral**	008.6, 008.8, 047, 070.0, 070.1, 070.6, 070.9, 074.3, 074.8, 079.0-079.3	A08.0, A0.8.2, A08.11, A08.19, A08.31, A08.32, A08.32, A08.39, A08.8, A87.0, A87.0, A87.8-A87.9, A88.8, B08.4, B15.0, B15.9, B19.0, B19.9, B33.8, B34.1, B34.9, B97.0, B97.10-B97.12, B97.89
**Unknown noninfectious**	558	K52.0-52.2, K52.81-K52.82, K52.89, K52.9
**Unknown infectious**	009	A09, B99.8-99.9, R19.7

### Analysis

We examined the trends in gastroenteritis-associated mortality using age-adjusted rates of mortality, calculated by direct standardization [16] with the 2000 U.S. population as estimated by the Census Bureau as the standard, for each pathogen category separately. Records for 1985–1998, which use ICD-9 codes, and 1999–2005, which use ICD-10 codes, were not directly comparable due to changes in coding and practices. Trends in annual age-adjusted mortality rates and percent of overall mortality due to gastroenteritis were assessed using a linear regression spline analysis to determine if there was a change in slope between the use of ICD-9 and ICD-10 coding.

The relative risks and 95% confidence intervals (CIs) for mortality due to gastroenteritis among all decedents in the U.S. between 1985 and 2005 were calculated using Poisson regression adjusted for age group, sex, race, and region. Analyses were conducted stratified by ICD usage.

All analyses were conducted using R software (version 2.14.1) and SAS software (version 9.2; SAS Institute, Cary NC).

## Results

There were 190,674 deaths related to gastroenteritis in the U.S. from 1985–2005 with an average of 9,080 per year. During this time the percent of deaths related to gastroenteritis more than tripled, increasing from 0.25% to 0.80% of all deaths (Table 1). There was a slight upward trend in the percent of deaths related to gastroenteritis in 1999; from 1985 to 1998 the percent of gastroenteritis-associated mortality increased by 0.0054% (β_pre1998_ = 0.0054 (95% CI: 0.0017, 0.0090)) annually and from 1999–2005 the annual increase was 0.0550% (β_post1999_ = 0.0550 (95% CI: 0.0154, 0.1146)). This increase may be due to the change in coding practices from ICD-9 to ICD-10. For 66.6% (127,046 of 190,674) of records gastroenteritis-associated conditions were listed as the underlying cause of death or in the first two contributing conditions (Table 2).

**Table 2:** **Prevalence and record placement of gastroenteritis as a cause of death**

**Year**	**Total no. of deaths related to GI**	**Total no. of deaths**	**% of deaths related to GI infection**	**Placement of diagnosis for gastroenteritis in mortality record deaths**					
				**Underlying cause**	**Contributing condition 1**	**Contributing condition 2**	**Contributing condition 3**	**Contributing condition 4**	**Contributing condition 5 or greater**
1985	5265	2089378	0.25	2229	2083	870	1007	716	637
1986	5386	2108384	0.26	2216	2107	886	1053	786	582
1987	5609	2126342	0.26	2295	2205	926	1118	800	588
1988	6054	2171196	0.28	2484	2280	988	1188	930	698
1989	6791	2153859	0.32	2776	2653	1165	1195	972	857
1990	6978	2151890	0.32	2744	2776	1154	1252	991	853
1991	7172	2173060	0.33	2839	2865	1320	1320	936	792
1992	7250	2179187	0.33	2775	2884	1359	1309	941	826
1993	7531	2271947	0.33	2742	3033	1376	1335	985	874
1994	7706	2282288	0.34	2839	3100	1469	1423	993	815
1995	7813	2315251	0.34	2969	3377	1417	1351	926	813
1996	7611	2318212	0.33	2945	3379	1409	1201	924	782
1997	7876	2317586	0.34	3202	3711	1346	1220	870	826
1998	8097	2340708	0.35	3289	3879	1379	1221	925	784
1999	9478	2394871	0.40	3865	5034	1306	1298	968	874
2000	10477	2407193	0.44	3734	5328	2018	1240	959	958
2001	10841	2419960	0.45	3959	5532	2133	1300	1015	889
2002	12655	2446796	0.52	4921	6955	2282	1378	1060	1024
2003	14029	2452154	0.57	5637	8349	2428	1306	961	1042
2004	16466	2401400	0.69	7026	10286	2735	1424	973	1116
2005	19589	2452506	0.80	8866	12515	3163	1650	1135	1233
1985-2005	190674	47974168	0.40						

**Figure 1:**
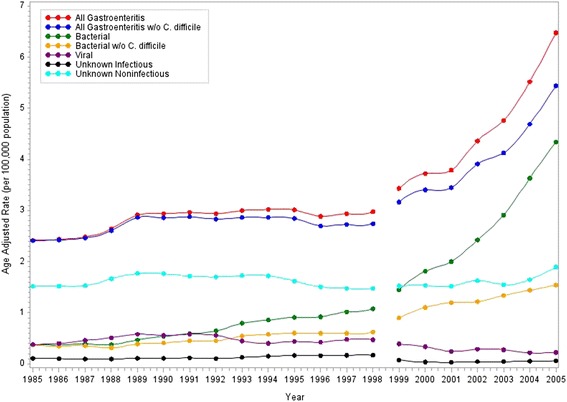
**Age adjusted rates of gastroenteritis-associated mortality by year (per 100,000).** The reference line at 1999 indicates when coding for mortality switched from ICD9 to ICD10.

The age adjusted rates demonstrate an increasing trend over time for overall mortality due to gastroenteritis and for most specific pathogen groups (Figure 1). Again we see an increase in rates in 1999 for overall mortality due to GI concurrent with the change in coding from ICD-9 to ICD-10 (Table 3). For all gastroenteritis-associated mortality there was an annual increase of 0.291 deaths per 100,000 from 1985 to 1998 which increased to 0.418 deaths per 100,000 annually from 1999 to 2005. GI deaths due to bacterial infections increases demonstrate a steep increase after 1999; from 1985 to 1998 there was an increase in age adjusted rates of bacterial gastroenteritis-associated deaths of 0.052 per 100,000 annually which increased to 0.428 deaths annually for the period from 1999 to 2005. However, this increase is less steep when cases of *C. difficile* are excluded, increasing from 0.032 per 100,000 annually before 1998 to 0.130 per 100,000 after 1999. Rates of viral and unknown infectious gastroenteritis-associated deaths both decrease over time (Table 3). There were no temporal trends observed in rates of unknown noninfectious gastroenteritis-associated deaths.

**Table 3:** **Trend analysis for age adjusted rates of gastroenteritis-associated mortality**

	**Slope from 1985 – 1998 β (95% CI)**	**Slope from 1999 – 2005 β (95% CI)**	**p-value (change in slope)**
**All gastroenteritis**	0.029 (0.001, 0.057)	0.418 (0.105, 0.883)	< 0.0001
**All gastroenteritis w/o** ***C. difficile***	0.016 (−0.006, 0.038)	0.316 (0.077, 0.676)	< 0.0001
**Bacterial**	0.052 (0.035, 0.069)	0.428 (0.099, 0.850)	< 0.0001
**Bacterial w/o** ***C. difficile***	0.032 (0.024, 0.039)	0.129 (0.052, 0.247)	< 0.0001
**Viral**	−0.003 (−0.010, 0.005)	−0.039 (−0.055, 0.018)	0.0031
**Unknown infectious**	0.003 (−0.001, 0.007)	−0.017 (−0.007, −0.048)	0.0011
**Unknown noninfectious**	−0.001 (−0.023, 0.004)	0.0229 (0.019, 0.092)	< 0.0001

Slopes (95% CI) for periods 1985 to 1998 and 1999 to 2005 and p-value for the change in slope between the two time periods.

Adjusted relative risks for gastroenteritis as a cause of death were calculated separately for years using ICD-9 codes, 1985–1998 (Table 4), and ICD-10 codes, 1999–2005 (Table 5). For 1985–1998, the elderly, those over 75 years, were at the highest risk of death due to gastroenteritis in all categories except unknown infectious gastroenteritis in which children 0–4 years of age were at the highest risk (RR = 46.25 (33.26, 64.32) compared to those on the referent age category of 35–44) (Table 4). During this time period, blacks had an almost 50% increased risk of gastroenteritis-associated mortality than whites (RR = 1.48 (1.24, 1.75)); this trend was consistent for all categories of gastroenteritis. Males also had higher risks of gastroenteritis-associated mortality for every grouping compared to females.

**Table 4:** **Adjusted relative risk (95%CI) for gastroenteritis as a cause of death in the US, 1985–1998 (ICD9)**

	**All gastroenteritis RR (95% CI)**	**All gastroenteritis w/o** ***C. difficile*** **RR (95% CI)**	**Bacterial RR (95% CI)**	**Bacterial w/o** ***C. difficile*** **RR (95% CI)**	**Viral RR (95% CI)**	**Unknown infectious RR (95% CI)**	**Unknown noninfectious RR (95% CI)**
**Age group**							
0-4	2.33 (1.66, 3.26)	2.37 (1.69, 3.31)	1.34 (1.03, 1.74)	1.48 (1.19,1.84)	0.88 (0.70, 1.10)	46.25 (33.26, 64.32)	1.68 (1.14, 2.48)
5-24	0.14 (0.11, 0.19)	0.15 (0.11, 0.19)	0.16 (0.12, 0.21)	0.18 (0.13, 0.23)	0.15 (0.12,0.19)	0.22 (0.14, 0.35)	0.13 (0.09, 0.18)
25-34	0.58 (0.40, 0.85)	0.58 (0.40, 0.85)	0.60 (0.44, 0.82)	0.61 (0.46, 0.81)	0.52 (0.40, 0.68)	0.50 (0.32, 0.78)	0.61 (0.37, 0.99)
35-44	referent	referent	referent	referent	referent	referent	referent
45-54	1.41 (1.03, 1.92)	1.41 (1.03, 1.92)	1.63 (1.25, 2.12)	1.65 (1.31, 2.08)	1.36 (1.05, 1.75)	1.67 (1.15, 2.44)	1.38 (0.96, 1.99)
55-64	3.02 (2.28, 3.99)	3.00 (2.27, 3.96)	3.62 (2.84, 4.61)	3.46 (2.83, 4.25)	2.36 (1.89, 2.94)	3.83 (2.74, 5.34)	3.36 (2.4, 4.71)
65-74	8.02 (6.10, 10.54)	7.86 (5.99, 10.33)	10.06 (8.07, 12.54)	8.70 (7.20, 10.51)	4.73 (3.88, 5.77)	9.76 (6.97, 13.67)	10.06 (7.20, 14.07)
75+	30.93 (23.34, 40.99)	29.99 (22.62, 39.76)	39.54 (31.60, 49.48)	29.88 (24.53, 36.40)	12.51 (9.99, 15.65)	32.67 (22.66, 47.11)	43.71 (31.15, 61.32)
**Race**							
White	referent	referent	referent	referent	referent	referent	referent
Black	1.48 (1.24, 1.75)	1.49 (1.26, 1.77)	1.67 (1.46, 1.91)	1.93 (1.72, 2.15)	1.45 (1.22, 1.72)	3.06 (2.47, 3.79)	1.23 (1.03, 1.48)
Other	0.77 (0.67, 0.88)	0.77 (0.68, 0.89)	0.86 (0.73, 1.03)	1.01 (0.83, 1.23)	1.07 (0.90, 1.28)	0.84 (0.67, 1.05)	0.56 (0.49, 0.65)
**Gender**							
Male	1.20 (1.10, 1.31)	1.20 (1.09, 1.31)	1.44 (1.34, 1.56)	1.55 (1.44, 1.67)	1.39 (1.25, 1.54)	1.23 (1.04, 1.45)	1.04 (0.96, 1.12)
Female	referent	referent	referent	referent	referent	referent	referent
**Region**							
Northeast	1.10 (0.96, 1.27)	1.07 (0.93, 1.22)	1.62 (1.42, 1.85)	1.47 (1.31, 1.63)	0.75 (0.63, 0.89)	1.03 (0.86, 1.22)	1.13 (0.99, 1.28)
Midwest	1.00 (0.87, 1.15)	0.99 (0.86, 1.14)	1.22 (1.12, 1.34)	1.25 (1.13, 1.39)	0.60 (0.52, 0.70)	1.02 (0.83, 1.25)	1.10 (0.96, 1.26)
South	1.04 (0.93, 1.18)	1.05 (0.93, 1.19)	1.44 (1.31, 1.57)	1.77 (1.57, 1.99)	0.71 (0.63, 0.79)	1.14 (0.94, 1.37)	1.04 (0.92, 1.18)
West	referent	referent	referent	referent	referent	referent	referent

**Table 5:** **Adjusted relative risk (95%CI) for gastroenteritis as a cause of death in the US, 1999–2005 (ICD10)**

	**All gastroenteritis RR (95% CI)**	**All gastroenteritis w/o** ***C. difficile*** **RR (95% CI)**	**Bacterial RR (95% CI)**	**Bacterial w/o** ***C. difficile*** **RR (95% CI)**	**Viral RR (95% CI)**	**Unknown infectious RR (95% CI)**	**Unknown noninfectious RR (95% CI)**
**Age group**							
0-4	2.00 (1.57, 2.53)	2.09 (1.67, 2.62)	0.78 (0.62, 0.98)	0.86 (0.72, 1.02)	2.94 (2.44, 3.54)	2.49 (1.14, 5.47)	3.90 (2.80, 5.45)
5-24	0.18 (0.15, 0.22)	0.18 (0.15, 0.23)	0.14 (0.11, 0.17)	0.14 (0.12, 0.16)	0.28 (0.23, 0.34)	0.29 (0.13, 0.66)	0.20 (0.15, 0.27)
25-34	0.43 (0.33, 0.55)	0.43 (0.34, 0.54)	0.44 (0.35, 0.55)	0.45 (0.38, 0.53)	0.40 (0.32, 0.51)	0.69 (0.29, 1.65)	0.42 (0.29, 0.60)
35-44	referent	referent	referent	referent	referent	referent	referent
45-54	2.22 (1.76, 2.81)	2.21 (1.77, 2.77)	2.58 (2.08, 3.20)	2.49 (2.20, 2.83)	1.90 (1.48, 2.43)	2.67 (1.40, 5.10)	2.14 (1.66, 2.75)
55-64	5.17 (4.24, 6.31)	4.99 (4.13, 6.03)	6.57 (5.45, 7.92)	5.57 (4.98, 6.24)	2.11 (1.71, 2.60)	7.01 (3.82, 12.86)	5.76 (4.58, 7.24)
65-74	14.44 (12.01, 17.35)	13.36 (11.21, 15.91)	18.40 (15.53, 21.82)	12.24 (10.98, 13.65)	3.30 (2.83, 3.85)	27.63 (15.52, 49.19)	17.79 (14.25, 22.21)
75+	61.72 (51.11, 74.55)	56.06 (46.76, 67.21)	75.98 (63.97, 90.26)	37.96 (34.10, 42.25)	11.72 (9.57, 14.36)	112.85 (63.63, 200.16)	83.16 (66.31, 104.29)
**Race**							
White	referent	referent	referent	referent	referent	referent	referent
Black	1.31 (1.13, 1.52)	1.37 (1.18, 1.59)	1.49 (1.30, 1.71)	2.39 (2.21, 2.58)	1.51 (1.25, 1.83)	1.09 (0.85, 1.38)	0.97 (0.81, 1.17)
Other	0.52 (0.47, 0.58)	0.55 (0.49, 0.61)	0.53 (0.46, 0.60)	0.75 (0.66, 0.84)	0.84 (0.71, 0.98)	0.31 (0.17, 0.56)	0.42 (0.38, 0.48)
**Gender**							
Male	1.06 (0.99, 1.13)	1.05 (0.98, 1.12)	1.16 (1.09, 1.23)	1.34 (1.27, 1.41)	1.30 (1.14, 1.49)	0.89 (0.81, 0.99)	0.87 (0.82, 0.93)
Female	referent	referent	referent	referent	referent	referent	referent
**Region**							
Northeast	1.22 (1.10, 1.34)	1.16 (1.05, 1.29)	1.42 (1.30, 1.56)	1.23 (1.12, 1.35)	0.82 (0.66, 1.03)	1.30 (1.12, 1.5)	1.02 (0.93, 1.12)
Midwest	1.18 (1.07, 1.30)	1.13 (1.01, 1.25)	1.27 (1.18, 1.37)	1.00 (0.94, 1.05)	0.86 (0.71, 1.04)	1.12 (0.95, 1.3)	1.13 (1.02, 1.26)
South	0.99 (0.92, 1.07)	0.98 (0.91, 1.06)	1.05 (1.00, 1.12)	0.98 (0.93, 1.02)	0.77 (0.68, 0.88)	1.08 (0.97, 1.2)	0.96 (0.88, 1.04)
West	referent	referent	referent	referent	referent	referent	referent

For the years using ICD-10 coding, 1999–2005, again the highest adjusted relative risks were seen in the oldest age category (Table 5) for all gastroenteritis pathogen groups. While children aged 0–4 years demonstrated a risk over twice has high as those aged 35–44 years (referent age category) (RR = 2.49 (1.14, 5.47)), this was much lower than the risk for this age group demonstrated during the years using ICD-9 coding. Though blacks had significantly higher risk of all gastroenteritis as a cause of death (RR = 1.31 (1.13, 1.52)), the risk of death related to an unknown pathogen was no higher than that of whites. Risk was significantly higher in males for bacterial and viral deaths (RR = 1.16(1.09, 1.23) and 1.34 (1.27, 1.41) respectively); however they were less likely to have unknown pathogen documented as a cause of death.

## Discussion

Over the 21-year period from 1985–2005 we estimated that an average of 9,080 deaths per year were due to gastrointestinal disease as an underlying cause or contributing factor. The number and percentage of deaths due to gastroenteritis demonstrated a steady increasing trend over the time period, even after adjusting for age. We demonstrated a significant increase in slope from a 0.0054% annual increase during the period 1985–1998, when ICD-9 coding was used, to a 0.0550% annual increase during 1999–2005, when ICD-10 coding was used. Though the analysis was limited by differing lengths of time for ICD-9 and ICD-10 usage, our analysis has shed light on the incompatibility between ICD-9 and ICD-10 coding practices for gastrointestinal diseases. ICD-10 coding requires an additional infection code to identify the specific pathogen as the cause of disease [17]. For some bacterial pathogens, such as streptococcus and staphylococcus, this pathogen specific code was not provided and these were grouped in broader bacterial categories. In addition, the guidance on the coding for unknown infectious and non-infectious GI varies from ICD-9 and ICD-10.

Gastroenteritis is typically considered a secondary infection and not the primary reason for hospitalization or death. However, we found gastroenteritis to be listed as the underlying cause of death or in the first two contributing conditions for the majority of records in both the ICD-9 and ICD-10 coding. The consistent increase over time and recording of gastroenteritis as a cause of death suggest an increase in coding and recognition of gastroenteritis as a cause of mortality by physicians. Improved diagnosis and testing methodology for gastroenteritis may also contribute to the increase in reporting of gastroenteritis-associated deaths.

The increase in gastroenteritis-associated mortality is primarily driven by an increase in reporting of bacterial infections. This trend remained even with the removal of the ICD codes for *Clostridium difficile* (ICD9: 008.45, ICD10: A04.7), which represented over 50% of deaths in the bacterial pathogen group. Recently, understanding the trends in gastrointestinal-associated mortality in the U.S. has been complicated due to the dramatic increase in *C. difficile*. Deaths due to *C. difficile*, primarily a nosocomial infection [18], contributed a majority of deaths and drove the results for bacterial-associated mortality, therefore trends were analyzed both with and without this cause. The increasing burden of mortality due to C. difficile has been well documented; with studies estimating a 5-fold increase due to *C. difficile* [4] and demonstrating an increase from 5.7 per million population in 1999 to 23.7 per million population in 2004 [19].

As expected, the elderly, 65 years and above, were at the highest risk of gastroenteritis-associated death for both the ICD-9 and ICD-10 coding periods. Gastroenteritis is often a complication of conditions which affect the elderly population, however, as noted, for the majority of cases gastroenteritis was indicated as the underlying cause or one of the primary causes of death. The aging population in the U.S. leads to more people with increased vulnerability to GI infections due to compromised immune systems [5,6,20]. We also found that blacks had a higher risk for death due to gastroenteritis compared to whites after adjustment for age. This increased risk maybe due delayed treatment of disease [21,22].

Our analysis has clinical implications as it demonstrates a lack of testing for specific gastrointestinal pathogens. Age adjusted rates for the unknown noninfectious disease category were higher than both the bacterial (w/o *C. difficle*) and viral categories. For 1985–1998, the youngest age group, 0–4 years, demonstrated the highest risk for the unknown infectious disease group. While this risk decreased for the years using ICD-10 coding it was still 2.5 times higher than the reference age group, 35–44 years. The ICD-10 time period also demonstrated a high relative risk, almost 4 times that of the reference group, for the unknown noninfectious disease group. Both the unknown infectious and unknown non-infectious disease groups are non-specific diagnosis codes which are used when specific pathogen testing has not been conducted. A study analyzing the seasonal patterns of these non-specific disease categories suggests that these may be due to viral pathogens [23]. However, increased awareness by clinicians of the need for laboratory testing for specific gastrointestinal pathogens would help to assess the mortality risk due to specific pathogens.

The viral pathogen group demonstrated a decreasing risk over time whereas the bacterial pathogen group demonstrated an increase over time. This increase was primarily driven by the bacterial pathogen, *C. difficile*. However, there was an increase even with the removal of the diagnosis code for *C. difficile* and maybe due to the increase in antibiotic resistant pathogens [24,25].

## Conclusion

In summary, the percent gastroenteritis-associated deaths tripled from 1985 to 2005. In 1999, ICD-10 coding was introduced for mortality and our analysis demonstrated a significant change in annual increase for the period using ICD-9 and that using ICD-10 coding. Those over 65 years of age were at the highest risk of reporting gastroenteritis as a cause of death for both coding practices. Overall, the black population is at the highest risk gastroenteritis-associated mortality. While mortality due to viral pathogens has been decreasing the deaths due to bacterial pathogens has increased. Gastroenteritis remains an important cause of mortality in the United States.

## Abbreviations

*C. difficile*: *Clostridium difficile*
GI: Gastrointestinal
ICD: International Classification of Diseases
ICD-9: International Classification of Diseases, Ninth Revision
ICD-10: International Classification of Diseases and Related Health Problems, Tenth Revision
